# Venetoclax and Daratumumab combination treatment demonstrates pre-clinical efficacy in mouse models of Acute Myeloid Leukemia

**DOI:** 10.1186/s40364-021-00291-y

**Published:** 2021-05-13

**Authors:** Jayna J Mistry, Charlotte Hellmich, Amelia Lambert, Jamie A Moore, Aisha Jibril, Angela Collins, Kristian M Bowles, Stuart A Rushworth

**Affiliations:** 1 Norwich Medical School, University of East Anglia, Norwich Research Park, NR4 7UQ Norwich, UK; 2 Earlham Institute, Norwich Research Park, NR4 7UH Norwich, UK; 3 Department of Haematology, Norfolk and Norwich University Hospitals NHS Trust, Colney Lane, NR4 7UY Norwich, UK; 4 Department of Molecular Haematology, Norwich Medical School, Norwich Research Park, NR4 7UQ Norwich, UK

## Abstract

Acute myeloid leukemia (AML) remains an incurable malignancy despite recent advances in treatment. Recently a number of new therapies have emerged for the treatment of AML which target BCL-2 or the membrane receptor CD38. Here, we show that treatment with Venetoclax and Daratumumab combination resulted in a slower tumor progression and a reduced leukemia growth both in vitro and in vivo. These data provide evidence for clinical evaluation of Venetoclax and Daratumumab combination in the treatment of AML.

**Supplementary Information:**

The online version contains supplementary material available at 10.1186/s40364-021-00291-y.

To the editor,

Acute Myeloid Leukemia (AML) is associated with poor prognosis and high mortality causing around 85,000 global deaths per year [1]. Current standard treatment for AML is poorly tolerated and most therapy is not curative for patients. The intensive nature of proficient therapeutics for AML are more suited towards younger patients, which is not the typical age-group for this disease. This results in limited effective treatment options, highlighting the need for more targeted, well-tolerated treatments.

Recently numerous novel therapies have emerged for treatment of AML. The BCL-2 family proteins are important in regulating intrinsic apoptotic processes [2]. Suppression of mitochondrial-mediated apoptosis by BCL-2 overexpression is a hallmark of AML progression, often correlated with a poor response to cytotoxic treatment [3, 4]. Recently, clinical trials using BCL-2 inhibition by the BH3 mimetic Venetoclax has been shown to be effective in promoting caspase-dependent AML cell death [5], leading to Venetoclax receiving FDA approval for treatment of AML in combination with low dose cytarabine, decitabine or the hypomethylating azacytidine [6]. This is an important milestone for AML treatment, as no curative therapies have shown to be clinically effective at treating this disease.

Unfortunately, AML resistance to Venetoclax is common, therefore, numerous studies combining Venetoclax with other therapies are being trialed [6, 7]. Our group and others have reported CD38 is overexpressed in AML and CD38 inhibition using Daratumumab is effective in preclinical AML models [8, 9]. CD38 is a transmembrane glycoprotein expressed on many hematopoietic cells [10]. In our recent study we demonstrated that Daratumumab works by inhibiting mitochondrial transfer from mesenchymal stromal cell (MSC) to AML blasts in the bone marrow (BM). Here we hypothesized that inhibiting the CD38 using daratumumab, in combination with BCL-2 inhibition using Venetoclax, could be a strategy to improve overall survival of AML patients.

To investigate BCL-2 inhibition in combination with CD38 inhibition in AML we used primary AML blasts isolated from patients BM (Table 1). CD38 and BCL-2 expression on isolated AML and non-malignant control CD34 cells were measured by flow cytometry (Fig. 1 a and supplementary Figure 1). Cell viability was assessed using various doses of Venetoclax or Daratumumab and showed that 100nM Venetoclax alone inhibited AML cell viability (Fig. 1b). Daratumumab alone had no effect on cell viability at any concentration used (Fig. 1b). Furthermore, in combination with Venetoclax, Daratumamab had no additive effect on AML survival (Fig. 1 c). Since AML is highly reliant on the BM microenvironment, we used an *in vitro* coculture system, AML was cultured with MSC with either Venetoclax alone, Daratumumab alone, or Venetoclax and Daratumumab combination for 24 h. AML cells were then stained with AnnexinV-FITC/PI and analyzed using flow cytometry. Cells treated with Venetoclax and Daratumumab combination had significantly higher apoptosis compared to untreated AML cells (Fig. 1d and e). Treatment with Venetoclax or Daratumumab alone had no significant effect on AML apoptosis when cultured under these conditions, suggesting that these targeted therapies are working through different pathways. Together, these results highlight the need to study the effectiveness of Venetoclax and Daratumumab combination in *in vivo* models of AML.

**Fig. 1:**
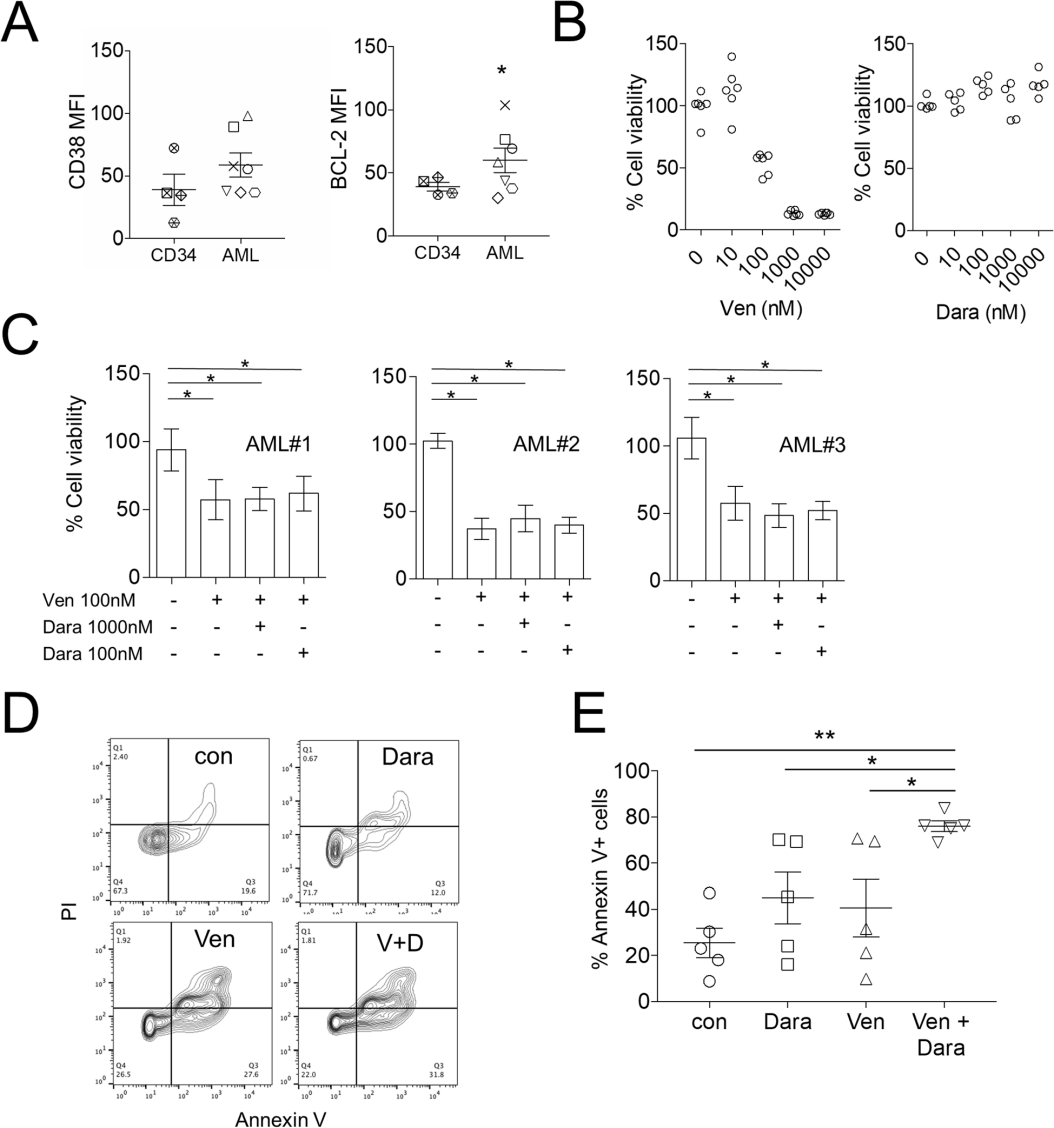
Venetoclax and Daratumumab inhibit AML cell growth. **a** Primary AML and CD34 + flow cytometry analysis for CD38 and BCL-2 expression as assessed by MFI (*n* = 7). **b** Primary AML (#1–6) were cultured together for 24 h in the presence of varying concentrations of Venetoclax alone or Daratumumab alone. Cell viability was assessed by CellTiter-Glo (*n* = 6) and expressed as a percentage compared to control untreated cells. **c** Primary AML were cultured together for 24 h in the presence of Venetoclax and varying concentrations of Daratumumab. Cell viability was assessed by CellTiter-Glo (*n* = 3). **d** and **e** Primary AML (#1, #2, #3, #7 and #9) and MSC cultured together for 24 h in the presence of vehicle, Venetoclax (100nm) alone or Daratumumab (100ng/ml) alone or Venetoclax and Daratumumab in combination. Primary AML were then stained with Annexin V/PI, flow cytometry was used to detect cell death (*n* = 5). Statistical tests used were Mann-Whitney U test (A) or Kruskal-Wallis statistical test followed by Dunn’s multiple comparisons (**b**-**e**). Data shown are means ± SD *P < 0.05 **P < 0.01

**Table 1:**
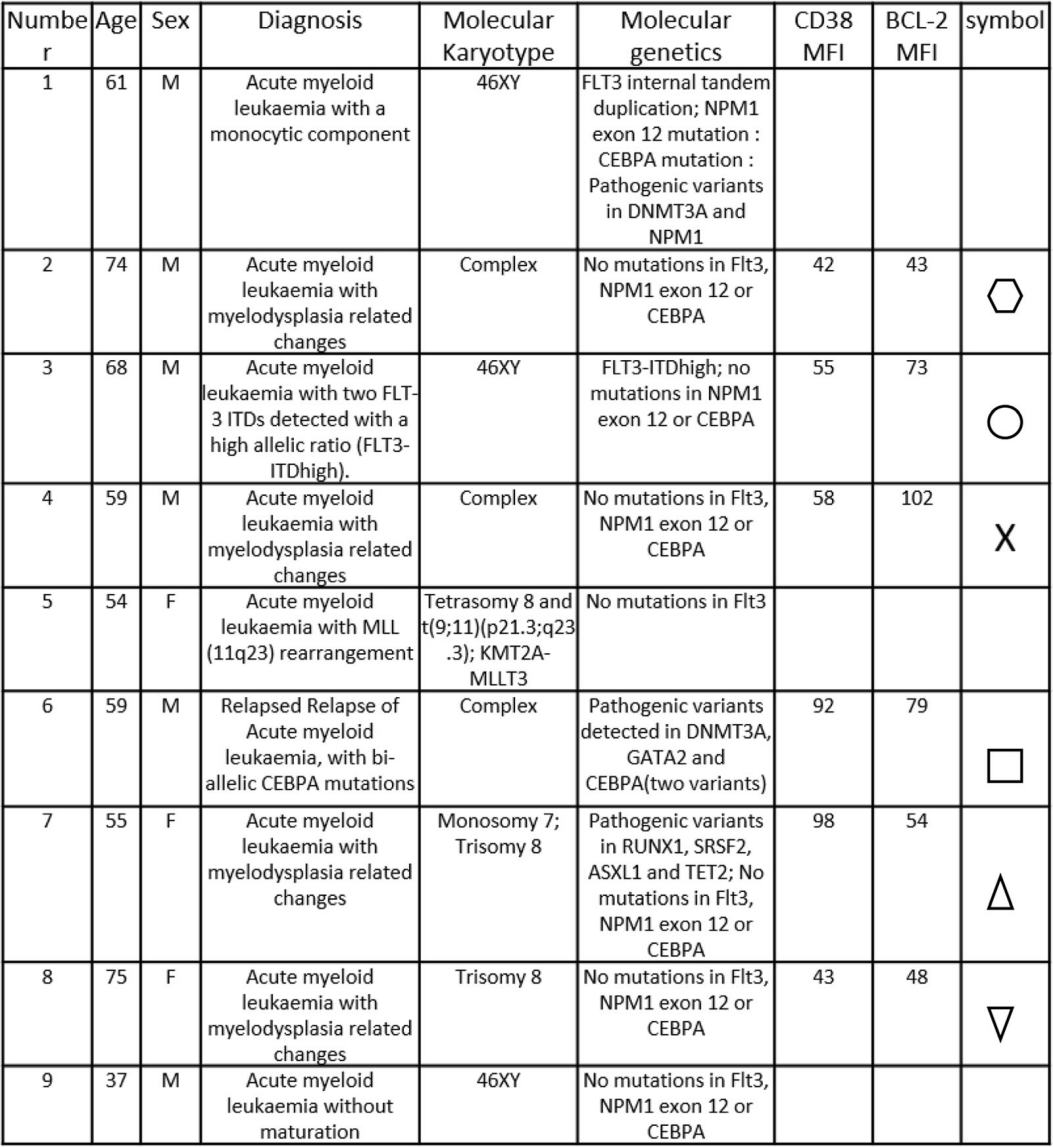
AML diagnostic information

To do this we used a NOD.Cg-Prkdcscid IL2rgtm1Wjl/SzJ (NSG) xenograft mouse model of AML whereby on day 1 we transplanted 0.5 × 10^6^ MV411 cells or patient derived AML tagged with a luciferase construct into the tail vein of NSG mice. On day 7 we analyzed NSG mice for AML engraftment. Mice were split into treatment groups including vehicle control (PBS), Daratumumab alone on day 7 and 14 by intraperitoneal injection (5 mg/kg), Venetoclax alone by oral gavage (100 mg/kg/day) for 10 days or combination Daratumumab (5 mg/kg) and Venetoclax (100 mg/kg/day) (Fig. 2 a). The mice were then imaged using live *in vivo* bioluminescence imaging on day 17 to determine tumor burden (Fig. 2b). The densitometry measurement of treated animals and survival graphs confirmed combination Daratumumab and Venetoclax was the most effective at reducing tumor burden (Fig. 2 c-f). Given the heterogeneity of AML, one limitation of the study is that only one AML patient sample and one cell line was used in the in vivo experiments. Finally, in phase 1/2 clinical trials in relapse/refractory myeloma combining Venetoclax with Daratumumab has a good safety profile [11].

**Fig. 2:**
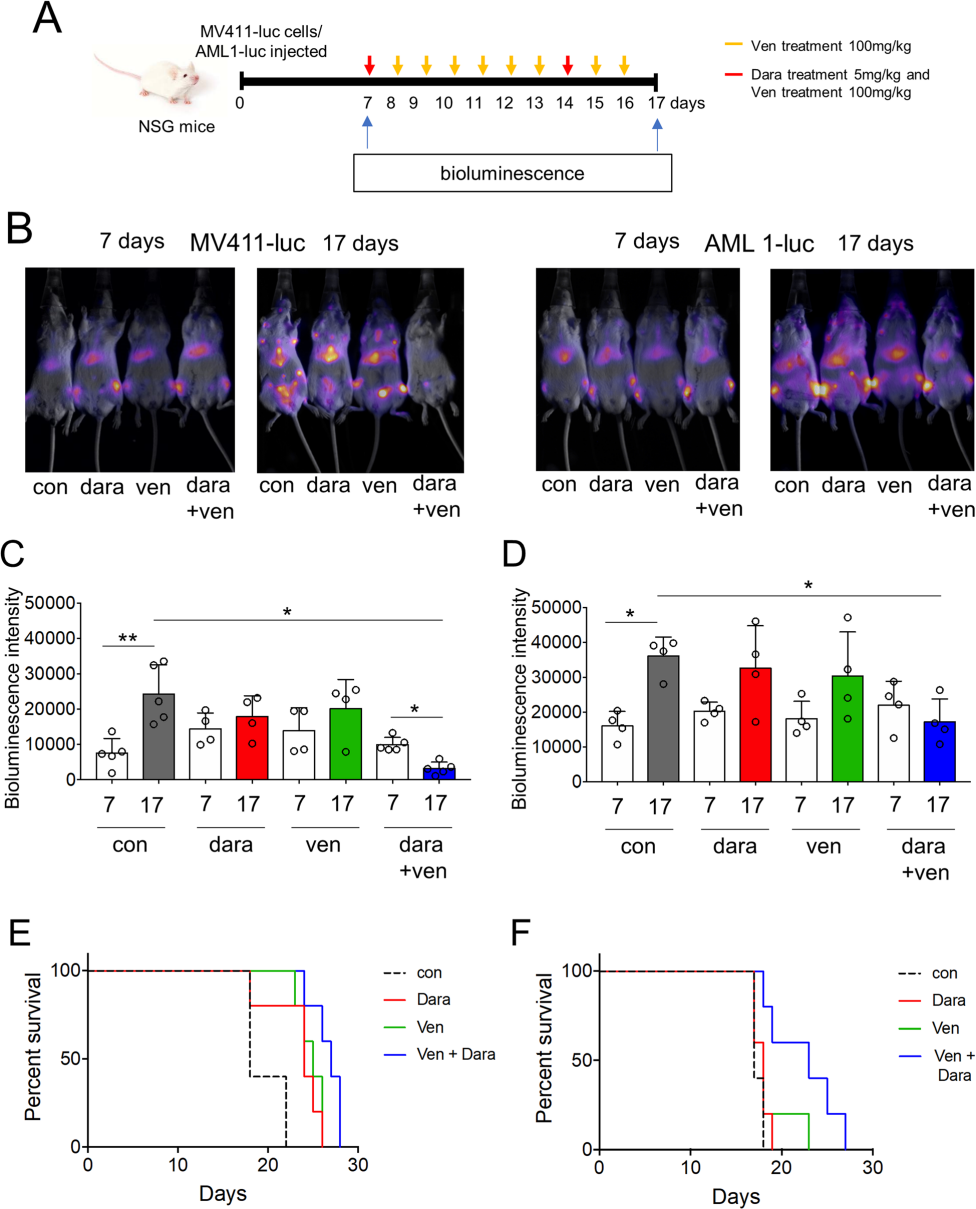
Venetoclax and Daratumumab combination inhibits AML disease progression in vivo. **a** Schematic of the *in vivo* model used for these experiments. **b** 0.5 × 10^6^ MV411 cells or primary AML (#3) were injected into the tail vein of NSG mice. Mice were imaged using bioluminescence at day 7 following injection to confirm tumor engraftment, and then split into four groups. Group 1 received vehicle (PBS), group 2 received Daratumumab (Dara; 5 mg/kg) on day 7 and day 14 by intra-peritoneal (IP) injection, group 3 received Venetoclax (Ven; 100 mg/kg) daily by oral gavage (OG) and group 4 received Venetoclax (Ven; 100 mg/kg) daily by OG and Daratumumab (Dara; 5 mg/kg) on day 7 and day 14 by IP. Mice were then imaged using bioluminescence at day 17. **c** and **d** Densitometry of the bioluminescent images shown in (B) was performed to determine differences between vehicle, Daratumumab alone, Venetoclax alone or Daratumumab and Venetoclax treated animals. (*n* > 4). Statistical tests used was Kruskal-Wallis statistical test followed by Dunn’s multiple comparisons. Data shown are means ± SD *P < 0.05 **P < 0.01. (E and F) Kaplan-Meier survival curves of treatment groups

Here, Venetoclax and Daratumumab combination treatment resulted in a reduced leukemia growth *in vitro* and *in vivo*. Our data opens a potential avenue for novel Venetoclax drug combination with Daratumumab and supports further clinical investigation as a therapeutic approach in the treatment AML.

## Supplementary Information

**Additional file 1: **

**Additional file 2: **

## Data Availability

All data generated or analysed during this study are included in this published article.

## Abbreviations

AML: Acute myeloid leukemia
BCL-2: B-cell lymphoma 2
BM: Bone marrow
IP: Intra-peritoneal
MSC: Mesenchymal stromal cells
NSG: NOD.Cg-Prkdcscid IL2rgtm1Wjl/SzJ
PBS: Phosphate buffered saline

## References

[1] Shafat MS, Gnaneswaran B, Bowles KM, Rushworth SA (2017). The bone marrow microenvironment - Home of the leukemic blasts. Blood Rev.

[2] Vaux DL (1998). Immunology. Ways around rejection. Nature.

[3] Tzifi F, Economopoulou C, Gourgiotis D, Ardavanis A, Papageorgiou S, Scorilas A (2012). The Role of BCL2 Family of Apoptosis Regulator Proteins in Acute and Chronic Leukemias. Adv Hematol.

[4] Campos L, Rouault JP, Sabido O, Oriol P, Roubi N, Vasselon C (1993). High expression of bcl-2 protein in acute myeloid leukemia cells is associated with poor response to chemotherapy. Blood.

[5] Pan R, Hogdal LJ, Benito JM, Bucci D, Han L, Borthakur G (2014). Selective BCL-2 inhibition by ABT-199 causes on-target cell death in acute myeloid leukemia. Cancer Discov.

[6] DiNardo CD, Pratz KW, Letai A, Jonas BA, Wei AH, Thirman M (2018). Safety and preliminary efficacy of venetoclax with decitabine or azacitidine in elderly patients with previously untreated acute myeloid leukaemia: a non-randomised, open-label, phase 1b study. Lancet Oncol.

[7] Ramsey HE, Fischer MA, Lee T, Gorska AE, Arrate MP, Fuller L (2018). A Novel MCL1 Inhibitor Combined with Venetoclax Rescues Venetoclax-Resistant Acute Myelogenous Leukemia. Cancer Discov.

[8] Naik J, Themeli M, de Jong-Korlaar R, Ruiter RWJ, Poddighe PJ, Yuan H (2019). CD38 as a therapeutic target for adult acute myeloid leukemia and T-cell acute lymphoblastic leukemia. Haematologica.

[9] Mistry JJ, Moore JA, Kumar P, Marlein CR, Hellmich C, Pillinger G, et al. Daratumumab inhibits acute myeloid leukaemia metabolic capacity by blocking mitochondrial transfer from mesenchymal stromal cells. Haematologica. 2020:haematol.2019.242974. 10.3324/haematol.2019.242974 PMC7849566 32193250

[10] Marlein CR, Piddock RE, Mistry JJ, Zaitseva L, Hellmich C, Horton RH (2019). CD38-Driven Mitochondrial Trafficking Promotes Bioenergetic Plasticity in Multiple Myeloma. Cancer Res.

[11] Kaufman JL, Baz RC, Harrison SJ, Quach H, Ho S-J, Vangsted AJ (2020). Updated analysis of a phase I/II study of venetoclax in combination with daratumumab and dexamethasone, +/- bortezomib, in patients with relapsed/refractory multiple myeloma. J Clin Oncol.

