# Supplementary figures and images for: Venetoclax and Daratumumab combination treatment demonstrates pre-clinical efficacy in mouse models of Acute Myeloid Leukemia
Source: Biomark Res. 2021 May 13;9:35. doi: 10.1186/s40364-021-00291-y (PMC8117650; doi:10.1186/s40364-021-00291-y)

## Slide 1
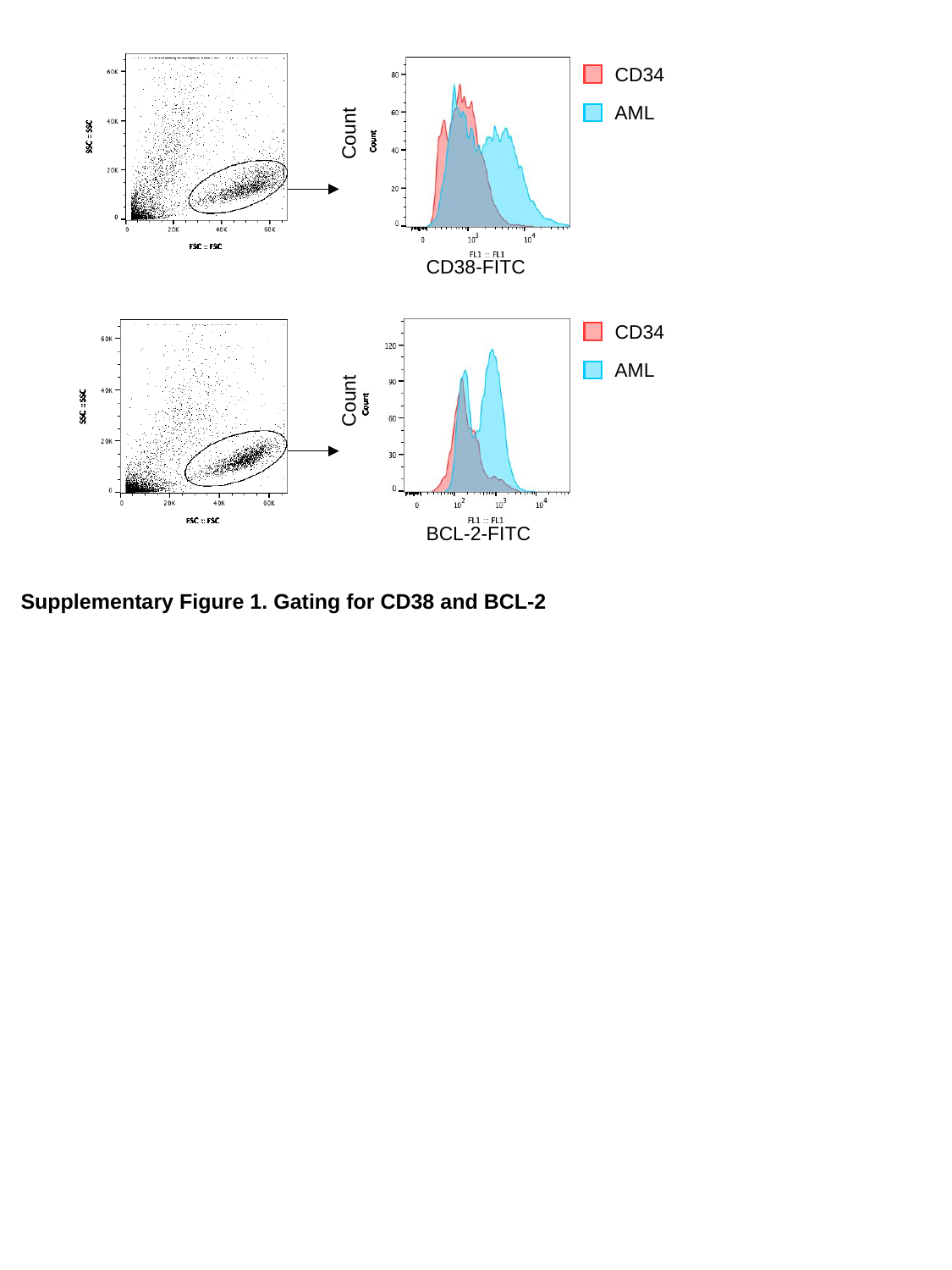

CD34
AML
Count
CD38-FITC
CD34
AML
Count
BCL-2-FITC
Supplementary Figure 1. Gating for CD38 and BCL-2

Supplement: Supplementary file 1 — Additional file 1: [file 40364_2021_291_MOESM1_ESM.pptx]
