# Supplementary material for: Venetoclax and Daratumumab combination treatment demonstrates pre-clinical efficacy in mouse models of Acute Myeloid Leukemia
Source: Biomark Res. 2021 May 13;9:35. doi: 10.1186/s40364-021-00291-y (PMC8117650; doi:10.1186/s40364-021-00291-y)
Supplement: Supplementary file 2 — Additional file 2: [file 40364_2021_291_MOESM2_ESM.pdf]

Methods

Animals

NOD.Cg-Prkdcscid IL2rgtm1Wjl/SzJ (NSG) mice (The Jackson Laboratory, Bar Harbour, ME, USA) were used in this study. $0.5x10^6$ primary AML blasts or MV411 AML cells were intravenously injected into non-irradiated NSG mice. Mice were monitored by *in vivo* bioluminescent imaging (Bruker, Coventry UK). Animals were housed in a specific pathogen-free facility. All animal work used in this study were carried out in accordance with regulations set by the UK Home Office and the Animal Scientific Procedures Act 1986. Mice used were at age 8-12 weeks of age and both genders were used for experiments.

Method Details

Primary cell isolation and culture

Primary AML blasts were obtained from the bone marrow of AML patients with informed consent, under approval from the UK National Research Ethics Service (LRCEref07/H0310/146). Non-malignant CD34+ haematopoietic stem cells were obtained from cord blood from caesarean section. AML cell isolation was carried out by gradient density centrifugation using Histopaque (Sigma-Aldrich) and cell type was confirmed by flow cytometry as previously described (1). CD34+ HSC were first isolated using gradient density centrifugation followed by CD34+ microbeads magnetic separation (Miltenyi Biotec). Mesenchymal stromal cell (MSC) were isolated by gradient density centrifugation and characterized by their fibroblast like shape and adherence to tissue culture plastic. MSC were expanded in Dulbecco’s Modified Eagle’s Medium (DMEM) supplemented with 20% foetal bovine serum (FBS) and 1% penicillin-streptomycin (Hyclone, Life Sciences). MSC were further characterised using flow cytometry for expression of CD90+, CD73+, CD105+ and CD45-.

Flow Cytometry

In this study the CyFlow Cube 6 (Sysmex, Milton Keynes, UK) was used. Cells were incubated for 5 minutes with an FCR receptor blocker (MIltenyi Biotec,) and then stained with isotype controls or test antibodies human CD38-FITC (Miltenyi Biotec). For experiments using BCL-2-FITC the cells fixed and permeabilized using the FIX &

PERM™ Cell Permeabilization Kit (ThermoFisher) as per manufacturer's instructions. The cells were then stained with BCL-2FITC, human and mouse (Miltenyi Biotec) for 20 minutes centrifuged at 1200rpm for 5 minutes before resuspending in PBS. Data were analyzed using FlowJo (TreeSta). See figures for specific gating strategies.

Annexin V assay

MSC were seeded at $5X10^4$ cells/well of a 24-well plate AML cells were placed on the MSC at a density of $1.5X10^5$ in normal growth media. Control PBS, Venetoclax (100nm) alone Daratumumab (100ng/ml) alone or Venetoclax and Daratumumab in combination were then added to the coculture. MSC/AML cocultures were then incubated for 24 hours. AML blasts were removed, and apoptosis was quantified using PI/AnnexinV assay (eBiosciences) and annexin V positive cells were measured using flow cytometry.

Cell titre glow

The viability of AML following culture with Daratumumab or Venetoclax was assessed using the CellTitre-Glo assay (Promeg). For the assay $1X10^4$ primary AML cells in 50μl were cultured in a 96 well plate and cultured with varying does of Daratumumab or Venetoclax or Venetoclax and Daratumumab in combination for 24hours. The cells were then transferred to a 96 well white plate and 50μl of CellTitre-Glo substrate was added and incubated for 10 minutes in the dark. Luminecence was measured at an emission wavelength 560nm using the LUMIstar Omega microplate reader (BMG LABTECH).

Bioluminescence imaging

To monitor AML engraftment bioluminescent imaging of live mice was carried out. Mice were intraperitoneally injected with150mg/kg D-luciferin on day 7 and 17. The mice were then left for 15 minutes at room temperature for maximum detection of the luciferase signal. The mice anaesthetised using a chamber filled with isoflurane. The mice were an imaged using a pre-set method of 1-minute exposure bioluminescent image, x-ray and light image.

Quantification and Statistical Analysis

Due to variability in the data, statistical comparison of work was performed without assumption of normal distribution using Mann-Whitney U test. For statistical comparison of more than two groups were compared, Kruskal-Wallis test followed by Dunn’s multiple comparisons using Prism version 7.00 for Windows (GraphPad). Differences among group means were considered significant when the probability value, p, was less than 0.05* 0.01**. Sample size (n) represents number of biological replicates. No statistical methods were used to predetermine sample size.

References

1. Zaitseva L, Murray MY, Shafat MS, Lawes MJ, MacEwan DJ, Bowles KM, et al. Ibrutinib inhibits SDF1/CXCR4 mediated migration in AML. Oncotarget. 2014;5(20):9930-8.
